# PbbHLH4 regulates floral monoterpene biosynthesis in *Phalaenopsis* orchids

**DOI:** 10.1093/jxb/ery246

**Published:** 2018-07-03

**Authors:** Yu-Chen Chuang, Yi-Chu Hung, Wen-Chieh Tsai, Wen-Huei Chen, Hong-Hwa Chen

**Affiliations:** 1 Department of Life Sciences, National Cheng Kung University, Tainan, Taiwan; 2 Institute of Tropical Plant Sciences, National Cheng Kung University, Tainan, Taiwan; 3 Orchid Research and Development Center, National Cheng Kung University, Tainan, Taiwan

**Keywords:** Floral scent, geranyl diphosphate synthase, monoterpenes, orchids, *Phalaenopsis*, transcription factors

## Abstract

Floral scent is an important factor in attracting pollinators and repelling florivores. In *Phalaenopsis bellina* (Orchidaceae), the major floral scent components are monoterpenoids. Previously, we determined that expression of GERANYL DIPHOSPHATE SYNTHASE (PbGDPS) is highly correlated with monoterpene biosynthesis in *Phalaenosis* orchids. Here, we found that both *cis*- and *trans*-regulation were present on the *GDPS* promoters, with *trans*-regulation playing a key role. To investigate the regulation of biosynthesis of floral scent, we compared the transcriptomic data of two *Phalaenopsis* orchids with contrasting scent phenotypes. Eight transcription factors (TFs) that exhibited sequential elevation in abundance through floral development in *P. bellina* were identified, and their transcript levels were higher in the scented orchid than the scentless one. Five of these TFs transactivated several structural genes involved in monoterpene biosynthesis including PbbHLH4, PbbHLH6, PbbZIP4, PbERF1, and PbNAC1. Ectopic transient expression of each of these TFs in scentless orchids resulted in stimulation of terpenoid biosynthesis. *PbbHLH4* most profoundly induced monoterpene biosynthesis, with a 950-fold increase of monoterpenoid production in the scentless orchid. In conclusion, we determined that biosynthesis of orchid floral monoterpenes was sequentially regulated, with PbbHLH4 playing a crucial role for monoterpene biosynthesis.

## Teaser

A basic helix-loop-helix transcription factor PbbHLH4 induces the biosynthesis of monoterpenes for floral scent in *Phalaenopsis* orchids.

## Introduction

Terpenoids, or terpenes, represent the largest group of plant floral volatiles (Tholl, 2015). They play important roles in attracting pollinators for successful reproduction (Blight *et al.*, 1997; Byers *et al.*, 2014) and in defense against pathogens and florivores (Junker *et al.*, 2011; Huang *et al.*, 2012). Apart from their natural roles, terpenoids are widely used in the cosmetics and perfume industry and as food additives because of their unique aromas and flavors (Schwab *et al.*, 2008; Caputi and Aprea, 2011).

The biosynthesis of terpene starts from the production of basic C5 units, isopentenyl diphosphate (IDP) and its isomer, dimethylallyl diphosphate (DMADP). Both C5 units are synthesized from the mevalonate (MVA) pathway in the cytosol or the methylerythritol phosphate (MEP) pathway in plastids. The C5 precursors derived from the MVA pathway are preferentially used for biosynthesis of sesquiterpenoids, and those generated by the MEP pathway are predominately used for monoterpenoids and diterpenoids. A group of enzymes called short-chain prenyltransferases are responsible for the successive condensation of IDP with DMADP to produce intermediates for terpene synthases (TPSs), including geranyl diphosphate synthase (GDPS) that produces geranyl diphosphate (GDP, C10) for monoterpenes, farnesyl diphosphate synthase that generates farnesyl diphosphate (FDP, C15) for sesquiterpenes, and geranylgeranyl diphosphate synthase that supplies geranylgeranyl diphosphate (C20) for diterpenes (Dudareva *et al.*, 2004).

*Phalaenopsis bellina* has a pleasant scent and is widely used as a breeding parent for its scent phenotype. The main floral scent volatiles in *P. bellina* are monoterpenoids, including linalool, geraniol, and their derivatives (Hsiao *et al.*, 2006). A combination of bioinformatics and genomics has highlighted *PbGDPS* as being enriched in the scented *P. bellina* but not in the scentless *P. equestris* (Hsiao *et al.*, 2006). Recombinant PbGDPS has dual prenyltransferase activities that produce GDP and FDP by using IDP/DMADP and IDP/GDP as substrates, respectively. *PbGDPS* shows flower-specific expression, and its highest expression is concurrent with maximal emissions of monoterpenoids on day 5 post-anthesis (D+5), which suggests a crucial role of PbGDPS in floral scent production in *P. bellina* (Hsiao *et al.*, 2008). Recently, we found that the emission of monoterpenes is under the control of light and a circadian rhythm in *Phalaenopsis* orchids (Chuang *et al.*, 2017).

Although our knowledge of the biosynthesis of floral terpenoids is increasing, little is known about its regulation. To date, the types of transcription factors (TFs) involved in terpene biosynthesis have been found to vary, and most studies on the regulation of terpene biosynthesis have been performed in vegetative tissues and fruit. Examples include AabHLH1, AabZIP1, AaERF1, AaERF2, AaNAC1, AaORA, and AaWRKY1 in *Artemisia annua* (Ma *et al.*, 2009; Yu *et al.*, 2012; Lu *et al.*, 2013; Ji *et al.*, 2014; Zhang *et al.*, 2015; Lv *et al.*, 2016), CitAP2.10 and CitERF71 in sweet orange (Shen *et al.*, 2016; Li *et al.*, 2017), CrBIS1, CrBIS2, CrBPF1, CrMYC2, CrORCA2-5, CrWRKY1, and CrZCT1-3 in *Catharanthus roseus* (van der Fits and Memelink, 2000; Pauw *et al.*, 2004; Suttipanta *et al.*, 2011; Zhang *et al.*, 2011; Li *et al.*, 2013, 2015*a*; Van Moerkercke *et al.*, 2015, 2016; Paul *et al.*, 2017), GaWRKY1 in cotton (Xu *et al.*, 2004), MsYABBY5 and MsMYB in spearmint (Wang *et al.*, 2016; Reddy *et al.*, 2017), NACs and EILs in kiwifruit (Nieuwenhuizen *et al.*, 2015), OsbZIP79, OsDPF, and OsTGAP1 in rice (Okada *et al.*, 2009; Miyamoto *et al.*, 2015; Yamamura *et al.*, 2015), SlMYC1, SlEOT1, and SlWRKY73 in tomato (Spyropoulou *et al.*, 2014*a*, 2014*b*), and ZmEREB58 in maize (Li *et al.*, 2015*b*). In contrast, regulation of floral terpene biosynthesis has been less well studied. To date, there has been only one study showing that AtMYC2 promotes inflorescence sesquiterpene production in Arabidopsis (Hong *et al.*, 2012). A summary of TFs known to regulate terpene biosynthesis is presented in Table S1 available at the Dryad Digital Repository, (https://doi.org/10.5061/dryad.kt056q7, Chuang *et al.*, 2018).

In this study, we aimed to elucidate the transcriptional regulation of floral scent biosynthesis by carrying out a comparative transcriptome analysis between scented and scentless orchids. Five TFs with higher expression in the scented orchid were identified, namely PbbHLH4, PbbHLH6, PbbZIP4, PbERF1, and PbNAC1. They showed a sequential expression pattern during floral development, which indicates developmental regulation. Ectopic transient expression of these TFs revealed that terpenoid biosynthesis in general was increased, with *PbbHLH4* strongly inducing monoterpene production in the scentless orchid. Taken together, the results broaden our knowledge of the regulating mechanisms controlling floral monoterpene biosynthesis. Many *Phalaenopsis* varieties and other modern floricultural cultivars have completely lost their scented phenotype as a result of traditional breeding methods, due to negative correlations between floral scent and other favorable traits (Vainstein *et al.*, 2001; Hsiao *et al.*, 2011). This study therefore provides a starting point for molecular breeding to introduce the scented trait in *Phalaenopsis* orchids.

## Materials and methods

### Plant material and growth conditions

Six *Phalaenopsis* orchids including two scented and four scentless ones that are commonly utilized as breeding parents were used in this study. The two scented orchids, *P. bellina* and *P.* Meidarland Bellina Age ‘LM128’, were purchased from Ming-Hui Orchids Nursery (Yunlin, Taiwan) and Meidarland Orchids (Tainan, Taiwan), respectively. The four scentless orchids were *P. javanica* (from Mi-Tuo Orchids, Kaohsiung, Taiwan), *P. mannii* (from Ji An Guang Feng, Hualien, Taiwan), and *P. aphrodite* subsp. *formosana* (hereafter abbreviated to *P. aphrodite*) and *P.* Sogo Yukidian ‘V3’ (from Taiwan Sugar Corp., Tainan, Taiwan). The scented orchid (*P. bellina*) has a much bigger genome than the scentless *P. aphrodite* (15.03 pg/2C versus 2.80 pg/2C) (Lin *et al.*, 2001). All plants were kept in a greenhouse at the National Cheng Kung University (NCKU, Tainan, Taiwan) under natural conditions.

### Chromatographic analysis of floral volatiles

The floral metabolites of the *Phalaenopsis* orchids were collected on day 5 post-anthesis (D+5) for 6 h (from 10.00 h to 16.00 h) as this represents their maximum emission interval (Chuang *et al.*, 2017). The flowers on the plants were placed in a scent-extracting apparatus as described by Chuang *et al.* (2017). To analyse the scent composition, metabolites were sampled from a single flower, with three biological replicates. The scent emission pattern of *P. bellina* was measured on a single flower from day 1 prior to anthesis (D–1) to D+23, with three biological replicates (senescence of *P. bellina* flowers usually occurs from D+25 to D+28). As a negative control, metabolites originating from the scent-extracting apparatus were analysed for background. The volatiles collected were eluted by hexane and identified by gas chromatography/high-resolution mass spectrometry (GC/HRMS) at the NCKU Instrument Center, as described by Hsiao *et al.* (2006, 2008).

### Transcriptome construction, assembly, and annotation

Transcriptomes were constructed for four floral stages of *P. bellina*: anthesis day (D0), D+3, D+5, and D+7. Total RNA was extracted from the entire flowers with duplicate biological repeats as described previously (Hsiao *et al.*, 2006, 2008). Quality control of RNA was performed using the RNA 6000 Nano Assay supplied with the Agilent 2100 bioanalyser. Preparation of four cDNA libraries and subsequent sequencing was carried out using an Illumina HiSeq 2000 at the Beijing Genomics Institute. *De novo* assembly for whole-transcriptome construction was carried out using the clean reads from the four libraries by using Trinity (release-20130225) (Grabherr *et al.*, 2011), and the resulting sequences were the unigenes. The expression level of each unigene in each sample was calculated as fragments per kilobase of transcript per million mapped reads (FPKM) based on the number of fragments uniquely aligned to the unigene in each library.

To gain insight into the function of the unigenes, the non-redundant (Nr) protein database at NCBI (https://www.ncbi.nlm.nih.gov/) was used to annotate them by using blastx with an *E*-value cut-off of 1.0 × 10^–5^. The floral transcriptome of *P. aphrodite* and its annotation were downloaded from Orchidstra (Su *et al.*, 2011, 2013*a*, 2013*b*), upgraded to Orchidstra 2.0 (http://orchidstra2.abrc.sinica.edu.tw/orchidstra2/index.php), in which the relative expression of unigenes is derived from microarray analyses at both the floral bud and full-blossom stages. Since two different methods were used to generate the *Phalaenopsis* transcriptome data, the most significant results were validated experimentally by quantitative real-time PCR.

### Identification of structural genes and TFs related to terpene biosynthesis

Three reference genes widely used in *Phalaenopsis* orchids were identified by using local blastn, namely *Actin4*, *Actin9*, and *Ubiquitin10*. We identified genes encoding enzymes related to monoterpene biosynthesis according to the KEGG annotation (https://www.genome.jp/kegg/annotation/). The genes isolated from the *P. bellina* floral transcriptome were confirmed by Nr annotation with an *E*-value cut-off of 1.0 × 10^–5^. The related genes in the *P. aphrodite* transcriptomes were identified by using local tblastx with an *E*-value cut-off of 1.0 × 10^–50^ and confirmed using the Nr annotation available on Orchidstra.

With the 181 TPS sequences collected from other plants as queries, *TPS* sequences of the two transcriptomes were isolated by using tblastx with an *E*-value cut-off of 1.0 × 10^–5^. The two transcripts of *PbTPS5* and *PbTPS10* were analysed. Since most monoterpene synthases (MTPSs) identified from other plants had an average peptide length of 600 residues, we focused on those with lengths close to this side, namely PbTPS5-1 (605 amino acids) and PbTPS10-2 (595 amino acids). The putative protein sequences of GDPS and four TPSs from both *P. bellina* and *P. aphrodite* are shown in Fig. S1 available at Dryad.

To isolate TFs and regulators, 28193 proteins in the PlnTFDB database (ver. 3.0; Pérez-Rodríguez *et al.*, 2010) were downloaded as queries to search the *P. bellina* transcriptome by using tblastn with an *E*-value cut-off of 1.0 × 10^–50^. The resulting sequences were then classified by using iTAK (Zheng *et al.*, 2016). Among them, 335 genes annotated as bHLH, bZIP, ERF, NAC, MYB, and WRKY were isolated, and 165 genes with FPKM>1 were imported into the Short Time-series Expression Miner (STEM) software (Ernst and Bar-Joseph, 2006) for classification of their expression profiles. For STEM analysis, the temporal expression profiles were transformed to start at 0 by subtracting the FPKM levels of the four floral stages (D0, D+3, D+5, and D+7) by the value for the first stage. The STEM Clustering Method provided by the software was used to cluster the factors into 10 profiles according to their expression patterns.

### Phylogenetic analysis

The full-length amino acid sequences of the putative TPSs isolated from the *P. bellina* and *P. aphrodite* transcriptomes (Su *et al.*, 2013*a*) were analysed phylogenetically with TPS sequences of other plant species. The MTPSs were predicted by the known TPSs in the same clade depending on the phylogenetic analysis (Fig. S2 at Dryad). For TF analysis, we downloaded basic helix-loop-helix (bHLH) TF families of Arabidopsis from The Arabidopsis Information Resource (TAIR, https://www.arabidopsis.org/index.jsp). The subgroup classification was analysed according to Heim *et al.* (2003). The MEGA6 software was used for alignment via ClustalW and the phylogenetic tree was built using the neighbor-joining method with 1000 bootstrap trials.

### Transactivation assay of the *GDPS* promoter in three *Phalaenopsis* orchids

Promoter fragments of *PbGDPS* were isolated by using a Universal GenomeWalker kit (Clontech, USA) and the primer design and PCR conditions for genome walking followed the manufacturer’s instructions (sequences of all primers used in this study are listed in Table S2 at Dryad). The 2.7-kb promoter fragment of *PbGDPS* was used for isolation of the *PaGDPS* promoter. We isolated a promoter fragment of *GDPS* from *P. bellina* and *P. aphrodite*, designated *PbGDPS*p for *P. bellina* and *PaGDPS*pA and *PaGDPS*pB for *P. aphrodite* because the sequencing results revealed that the promoter fragments of *PaGDPS* were polymorphic with two types based on the deletion region. The 1076-, 1065-, and 1028-bp fragments of *PbGDPS*p, *PaGDPS*pA, and *PaGDPS*pB, respectively, were cloned into pJD301(f) to drive the firefly luciferase gene. In addition, an internal control pJD301(R) was included, which contained the *Renilla* luciferase gene driven by the cauliflower mosaic virus (CaMV) 35S promoter. The plasmids harboring promoter fragments and the internal control were then co-bombarded into *Phalaenopsis* floral tissues at a ratio of 10 to 0.1 μg as described previously (Hsu *et al.*, 2014). Bombardment of flowers of *P. bellina* and *P. aphrodite* at the D+5 stage was carried out with three replicates. The luciferase activity of each sample was measured after 20 h of incubation. The tissues were ground in liquid nitrogen using a pestle and mortar, and the resulting fine powder was dissolved in the PLB solution provided with the Dual-Luciferase Reporter (DLR) Assay System (Promega). The luciferase activity was determined according to the manufacturer’s instructions. Pairwise comparisons between groups were performed using Tukey’s honestly significant difference test at α=0.05.

### Quantitative real-time PCR

Total RNA was extracted as described previously (Hsiao *et al.*, 2006, 2008). For *P.* Meidarland Bellina Age ‘LM128’, *P. aphrodite*, *P. javanica*, and *P. mannii*, total RNA was extracted at the floral D+5 stage. For *P. bellina* total RNA was extracted at five floral stages: D–1, D0, D+3, D+5, and D+7. After removal of DNA contamination by DNase (NEB, UK), the RNA samples were reverse-transcribed to cDNA using SuperScript III (ThermoFisher Scientific). Primers were designed to detect transcripts of *P. bellina* and *P. aphrodite* simultaneously based on the corresponding transcripts in the two orchid transcriptomes. Quantitative real-time PCR was carried out using a StepOnePlus Quantitative Real-Time PCR System and a SYBR Green kit (Applied Biosystems) as described previously (Hsu *et al.*, 2015). The expression of all genes was normalized to the reference gene, *PbActin1*. Three biological replicates were used, and pairwise comparisons between groups were performed using Tukey’s honestly significant difference test at α=0.05.

### Examination of the transactivation of TFs on the promoters of structural genes

Promoter fragments of *PbGDPS2*, *PbTPS5*, and *PbTPS10* were isolated from genomic DNA of *P. bellina* by using primers designed according to the *P. equestris* draft genome (Cai *et al.*, 2015). After sequence confirmation, 1175-, 887-, and 1016-bp of promoter fragments for *PbGDPS2*, *PbTPS5*, and *PbTPS10*, respectively, were cloned into pJD301(f) to drive the firefly luciferase gene. The coding sequences for *PbbHLH2*, *PbbHLH4*, *PbbHLH5*, *PbbHLH6*, *PbbZIP4*, *PbERF1*, *PbMYB22*, and *PbNAC1* were amplified from full-bloom flowers of *P. bellina* using gene-specific primers and cloned into pBI221 to replace the *GUS* (β-glucuronidase) gene and were driven by the CaMV 35S promoter. Three separate plasmids—pBI221 containing TFs, pJD301(f) containing promoter fragments (four sequences, together with the *PbGDPS*p), and the internal control pJD301(R)—were co-bombarded into *P. aphrodite* floral tissues at a ratio of 1.5:1.5:0.15 (total 3.15 µg) as described previously (Hsu *et al.*, 2014). The luciferase activity of each sample was measured after 20 h of incubation. The relative fold-change in activity was calculated by comparison with the control assay for *GUS* in pBI221. The assays with TFs were performed with three biological repeats. The numbers of replicates for native promoter activity without TFs (with GUS instead) were as follows: *PbGDPS*p, *n*=27; *PbGDPS2*p, *n*=12; *PbTPS5*p, *n*=15; and *PbTPS10*p, *n*=18. Several combinations showing variance among replicates were performed with six replicates to obtain more reliable results: PbbHLH2, PbbHLH6, and PbNAC1 for *PbGDPS*p. Pairwise comparisons between groups were performed by using Tukey’s honestly significant difference test at α=0.05.

### Transient ectopic expression of TFs in orchids

Plasmids were constructed as described previously (Hsu *et al.*, 2015). The coding sequences of *PbbHLH4*, *PbbHLH6*, *PbbZIP4*, *PbERF1*, and *PbNAC1* were amplified from full-bloom flowers of *P. bellina* using gene-specific primers and transferred to the vector p1304NhXb under a duplicated CaMV 35S promoter. *Agrobacterium tumefaciens* EHA105 carrying the resulting clones was infiltrated into scentless flowers of *P. aphrodite* and *P.* Sogo Yukidian ‘V3’ on the day of anthesis (D0) with three replicates as described previously (Hsu *et al.*, 2015). The promoter containing GUS only was used as the negative control. The metabolites emitted from infiltrated flowers were collected at 4 d post-infiltration (DPI) for 8 h (from 8.00 h to 16.00 h) as described above, and the compounds were identified by GC/HRMS as described by Hsiao *et al.* (2006, 2008). Total RNA was extracted at 5 DPI as described above, and gene expression analysis was performed by quantitative real-time PCR. Statistical analysis was performed using Student’s *t*-test at α=0.05.

### Accession numbers

Sequence data supporting the findings of this study have been deposited in GenBank under accession numbers PbGDPS (EU023907) and PbbHLH4 (KY979199).

## Results

### Profiles of floral volatile in scented *P. bellina* and scentless *P. aphrodite*

**Fig. 1.**
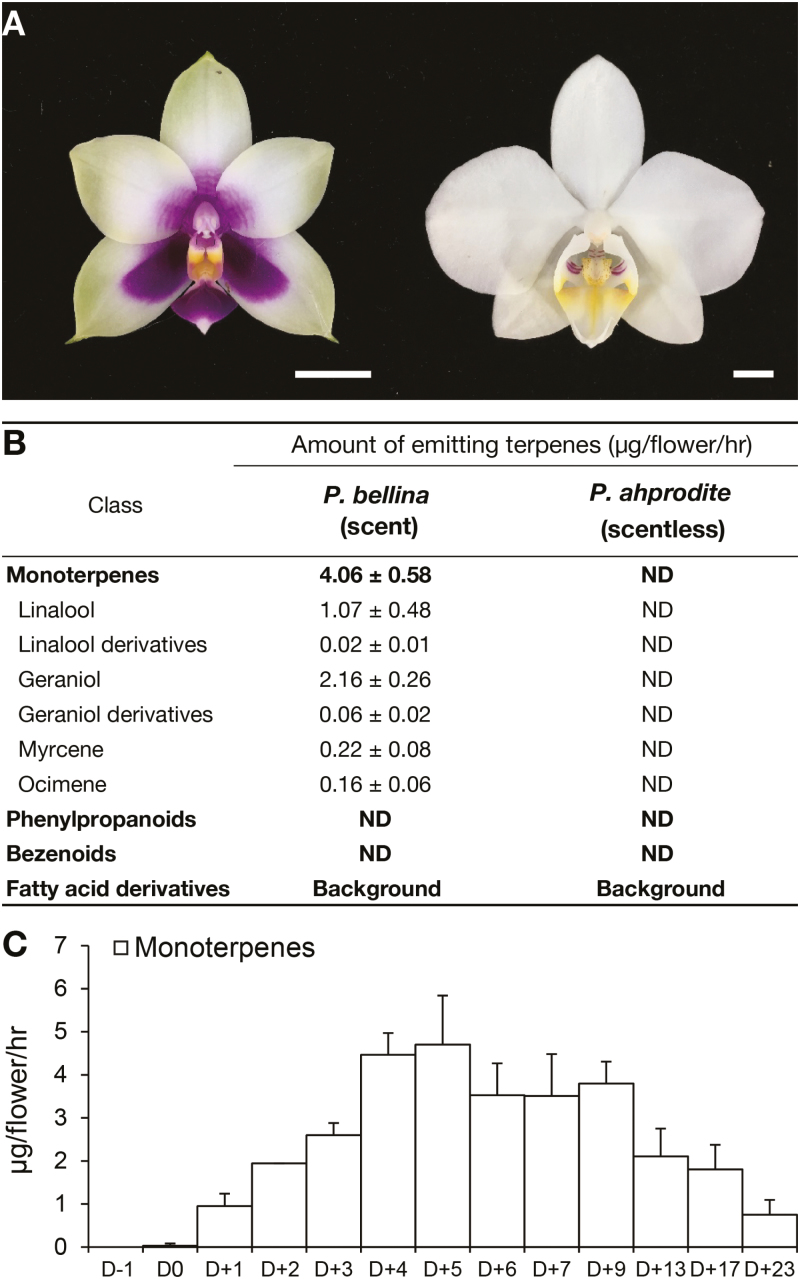
Floral volatile profiles of *Phalaenopsis bellina* and *P. aphrodite*. (A) Single flowers of *P. bellina* (left) and *P. aphrodite* (right). Scale bars are 1 cm. (B) Floral volatile profiles of *P. bellina* and *P. aphrodite* at the full-bloom stage, 5 d after anthesis (D+5). Data are means (±SE) of three replicates. ND, not detected. ‘Background’ indicates that the fatty-acid derivatives detected were emitted from the scent-extracting apparatus. (C) The emission pattern of total monoterpenes from the day before anthesis (D–1) to D+23 in *P. bellina*. Data are means (±SE) of three replicates.

To study the regulation mechanism of floral scent biosynthesis in *Phalaenopsis* orchids, we selected two native species with contrasting scent profiles, *P. bellina* (scented) and *P. aphrodite* (Fig. 1A), which are important parents in breeding programs for scent and white-color traits, respectively. In addition, *P. aphrodite* is a commonly cultivated and commercialized *Phalaenopsis* species. We first checked the volatiles emitted from a single flower of the two species (Fig. 1B). Two monoterpenoids, linalool and geraniol and their derivatives, accounted for 25% and 50%, respectively, of the total amount of floral volatiles in *P. bellina*. In contrast, no scent compounds were detected in *P. aphrodite*. The emission pattern of monoterpenes throughout the floral developmental stages was then further examined in *P. bellina*. Monoterpenoids were absent in floral buds (D–1), were initiated on the day of anthesis (D0), rapidly increased in emissions during flower maturation, peaked at the full-bloom stages (D+4 and D+5), and gradually decreased thereafter with flower age (Fig. 1C). Thus, production of monoterpenoids was developmentally co-ordinated during the flowering of *P. bellina*.

### Key steps in monoterpene biosynthesis in *Phalaenopsis* orchids

**Fig. 2.**
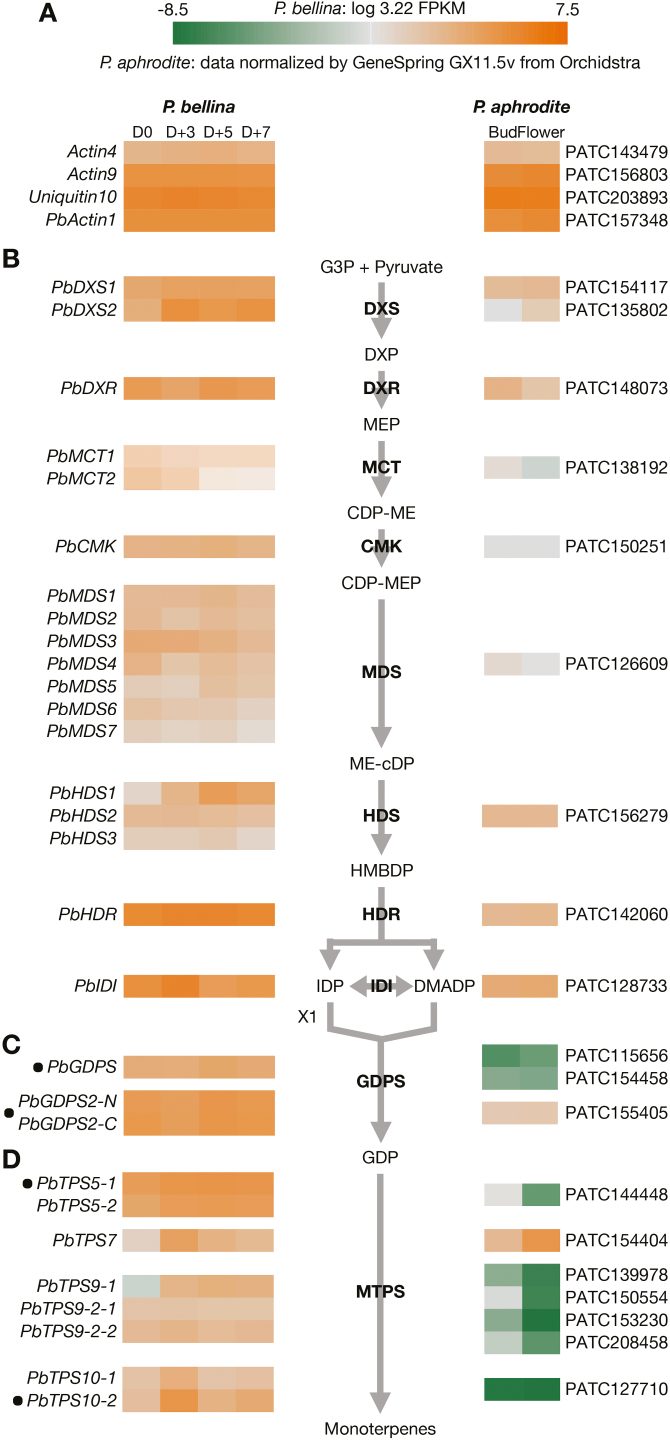
Comparative expression profiles of putative genes encoding enzymes for monoterpene biosynthesis from two *Phalaenopsis* transcriptomes. (A) The putative gene expression in *P. bellina* and *P. aphrodite* transcriptomes is represented by a color gradient from orange to green. The reference genes and their homologs in *P. aphrodite* were included. (B) Expression of genes encoding enzymes in the MEP pathway. The abbreviated names of enzymes in each catalytic step are in bold. Putative genes are organized according to their annotated function. Isopentenyl diphosphate isomerase (IDI) is the enzyme that catalyses the isomerization between IDP and DMADP. (C) Expression of *GDPS* and *GDPS2* in both transcriptomes. *PbGDPS2-N* and *PbGDPS2-C* indicate the N and C termini of *PbGDPS2*, respectively. (D) Expression of putative genes annotated as *MTPS*s in both transcriptomes. Genes that were further analysed by transactivation assays *in planta* are labeled with black dots. The homologous genes between two *Phalaenopsis* orchids are aligned opposite each other. Abbreviations for enzymes or compounds not described in the article are as follows: CDP-ME, 4-diphosphocytidyl-2-C-methylerythritol; CDP-MEP, 4-diphosphocytidyl-2-C-methyl-D-erythritol 2-phosphate; DXP, 1-deoxy-D-xylulose 5-phosphate; DXR, 1-deoxy-D-xylulose-5-phosphate reductoisomerase; G3P, glyceraldehyde-3-phosphate; IDI, isopentenyl diphosphate isomerase, HMBDP, 4-hydroxy-3-methyl-but-2-enyl pyrophosphate; ME-cDP, 2-C-methyl-D-erythritol 2,4-cyclodiphosphate.

To explore the mechanisms underlying the contrasting monoterpene biosynthesis of the two orchids, the floral transcriptomes of *P. bellina* and *P. aphrodite* were compared. The transcriptomic data of *P. bellina* were constructed using floral material at four developmental stages (D0, D+3, D+5, and D+7) that represented the four phases of the monoterpene emission pattern: onset, increase, peak, and decline (Fig. 1C). In addition, floral transcriptomic data for *P. aphrodite* were downloaded from Orchidstra (Su *et al.*, 2011, 2013*a*), which contains information for both the floral bud and full-blossom stages. The transcript abundance of the *P. bellina* transcriptome was determined by using FPKM, and the gene expression of *P. aphrodite* was analysed by microarray assays (Su *et al.*, 2013*b*). To compare the transcriptomic profiling between the two different platforms, the FPKM values of the *P. bellina* transcriptome were transformed by log_3.22_. After this transformation, the expression of three reference genes that are typically analysed in *Phalaenopsis* orchids were found to exhibit equivalent levels between the RNA-seq and microarray assay data: *Actin4* (Chen *et al.*, 2005; Hsieh *et al.*, 2013; Pan *et al.*, 2014; Hsu *et al.*, 2015), *Actin9* (Hsiao *et al.*, 2008; Pan *et al.*, 2011, 2014; Hsu *et al.*, 2015), and *Ubiquitin10* (Lu *et al.*, 2007; Hsiao *et al.*, 2008) (Fig. 2A). In addition, *Actin1*, which had identical expression between the two transcriptomes (Fig. 2A), was used as an internal calibrator for further analysis. The expression levels of these reference genes in both transcriptomes are available at Dryad (see Dataset 1, ‘Reference’ spreadsheet, https://doi.org/10.5061/dryad.kt056q7).

The putative genes encoding each step of the MEP pathway were further identified in both orchids. Expression of these genes, which are responsible for the biosynthesis of IDP and DMAPP, did not differ much between the two orchids (Fig. 2B), although the putative genes encoding 2-C-methyl-D-erythritol 4-phosphate cytidylyltransferase (MCT), 4-(cytidine 5’-diphospho)-2-C-methyl-D-erythritol kinase (CMK), 2-C-methyl-D-erythritol 2,4-cyclodiphosphate synthase (MDS), and 1-hydroxy-2-methyl-2-(E)-butenyl 4-diphosphate reductase (HDR) were slightly down-regulated in *P. aphrodite*. In addition, multigene families identified for several steps in the MEP pathway for *P. bellina* included 1-deoxy-D-xylulose 5-phosphate synthase (DXS), MCT, MDS, and 4-hydroxy-3-methylbut-2-enyl diphosphate synthase (HDS).

Intriguingly, in addition to the original *PbGDPS* (27 kDa) (Hsiao *et al.*, 2008), another gene encoding geranyl diphosphate synthase (57 kDa) was also identified and designated as *PbGDPS2*. We detected significant differential expression of *GDPS* between *P. bellina* and *P. aphrodite* whereas little or no differences were observed for the expression of *GDPS2* (Fig. 2C). In addition, among four putative genes encoding monoterpene synthases (*MTPS*s) (Fig. S2 at Dryad), three were more significantly up-regulated in *P. bellina*, namely *PbTPS5*, *PbTPS9*, and *PbTPS10* (Fig. 2D), consistent with its monoterpene production. *TPS7* was expressed at similar levels in both orchids. Therefore, we concluded that the enhanced expression of *GDPS* and *MTPS*s may have accounted for the monoterpene biosynthesis in the orchids. *GDPS* was the first enzyme in the pathway with significant differential expression, and it provides the precursors for further monoterpene biosynthesis. Hence, the absence of elevated *GDPS* expression in the scentless *P. aphrodite* might be responsible for the lack of monoterpene accumulation. The expression levels of these structural genes in both transcriptomes are available at Dryad (see Dataset 1, ‘Enzyme’ spreadsheet, https://doi.org/10.5061/dryad.kt056q7).

### *trans*-factors are critical for *GDPS* expression

**Fig. 3.**
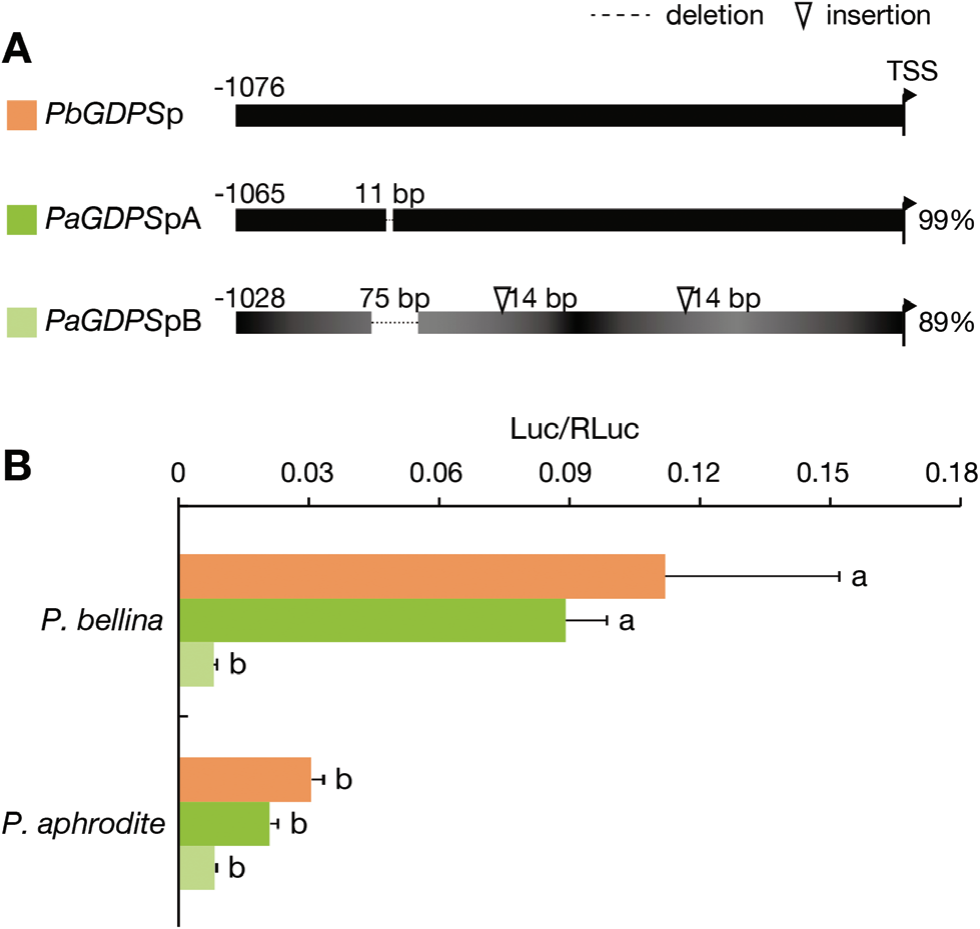
*GDPS* promoter activities *in planta*. (A) Sequence differences of three *GDPS* promoter fragments isolated from *P. bellina* (*PbGDPS*p) and *P. aphrodite* (*PaGDPS*pA and *PaGDPS*pB). The numbers indicate base-pairs upstream of the translational start site (TSS). Dotted lines and triangles indicate large-fragment deletions and insertions, respectively, in the two promoter fragments of *PaGDPS* as compared to *PbGDPS*p. Comparative similarities are indicated on the right. The numerous substitutions of *PaGDPS*pB are indicated by the varying shading. (B) Comparative activities of three promoter fragments in the floral tissues of the two *Phalaenopsis* species as determined by dual-luciferase assays. The activation level is given by the ratio of Luc/RLuc. Data are means (±SE) of three biological replicates. Pairwise comparisons between groups were performed using Tukey’s honestly significant difference test, and different letters indicate significant differences at α=0.05.

These results prompted us to investigate the mechanisms underlying the absence of *GDPS* expression in the scentless *P. aphrodite*. We isolated a 1-kb promoter fragment of *GDPS* from *P. bellina* and *P. aphrodite*, designated *PbGDPS*p for *P. bellina* and *PaGDPS*pA and *PaGDPS*pB for *P. aphrodite* because two promoter fragments were isolated. *PaGDPS*pA shared 99% identity with *PbGDPS*p, with an 11-bp deletion between nucleotides –836 to –823 (Fig. 3A). *PaGDPS*pB shared 89% identity with *PbGDPS*p, with a 75-bp deletion (nucleotides –859 to –785), two 14-bp insertions at nucleotides –763 and –355, and numerous nucleotide substitutions.

To assess the activity of these three promoter fragments, we particle-bombarded live perianths of scented *P. bellina* and scentless *P. aphrodite* flowers with various constructs for dual luciferase assays (Fig. 3B). Both *PbGDPS*p and *PaGDPS*pA activities were substantially greater in scented than scentless flowers, so the activators for both promoters were up-regulated in the scented *P. bellina* but down-regulated in the scentless *P. aphrodite.* In contrast, we detected no differential luciferase activity for *PaGDPS*pB in scented or scentless plants, which suggested impaired promoter activity of *PaGDPS*pB associated with its deletions, insertions, or nucleotide substitutions. These results implied that both *trans*-factors and *cis*-elements are required for the *GDPS* promoter activity, although *trans*-factors played the major role.

### Correlation analysis to identify eight TFs associated with *PbGDPS* expression

Several types of TFs for terpene biosynthesis have been detected in various plant species, including bHLH, bZIP, ERF, NAC, MYB, and WRKY. A total of 335 TFs categorized in these types were identified in the *P. bellina* transcriptome by a BLAST search of PlnTFDB (Pérez-Rodríguez *et al.*, 2010) and classified by iTAK (Zheng *et al.*, 2016). Because *PbGDPS* was highly expressed in the *P. bellina* transcriptome (FPKM>30), we selected 165 TF genes showing FPKM values >1 for further analysis. We applied two criteria to identify candidate TFs regulating *GDPS* for monoterpene biosynthesis: (1) the TFs had to appear either prior to or concurrent with the expression pattern of *PbGDPS* during flower development, and (2) the expression of the TFs had to be up-regulated in *P. bellina* but down-regulated in *P. aphrodite*.

**Fig. 4.**
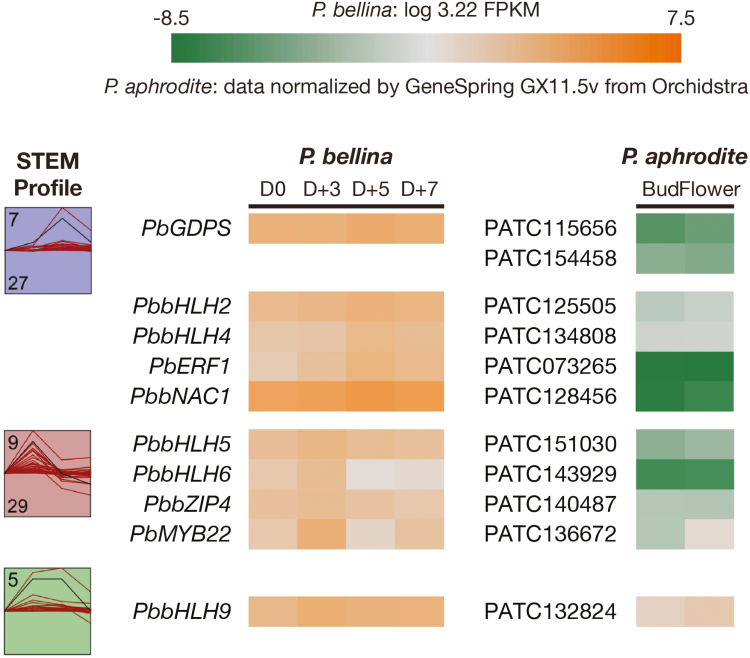
Bioinformatics analysis of transcription factors (TFs) to identify candidates. Two STEM profiles, No. 7 and No. 9, were selected for identifying candidate TFs in *P. bellina* transcriptomes (see Methods). Expression is represented by a color gradient from orange to green. The four time points represent the FPKM values at four floral developmental stages (day of anthesis D0, D+3, D+5, and D+7), which were normalized and transformed to set the time series to start at 0 (Fig. S2 at Dryad). The STEM profiles include the profile number at the top-left and the number of genes at the bottom-left. The model expression pattern of each profile is displayed as a black line, while the red lines are for individual genes. PbbHLH9, a bHLH TF belonging to STEM profile No. 5, is also included.

In the first screening, clustering by STEM (Ernst and Bar-Joseph, 2006) was performed to associate the expression patterns of the 165 TFs with that of *PbGDPS*, and 10 distinct STEM profiles (labelled 0 to 9) were generated (see Dataset 2 for full clustering results and see Fig. S3 at Dryad). *PbGDPS* showed maximal expression on D+5 and was classified into profile ‘7’, together with 26 TFs with concurrent expression (Fig. 4). In addition, 29 TFs in profile ‘9’ were selected for their expression prior to *PbGDPS*, which peaked on D+3. In the second screening, the expression of 55 TFs was compared to that of the scentless *P. aphrodite*. Among them, eight candidate TFs, namely *PbbHLH2*, *PbbHLH4*, *PbHLH5*, *PbbHLH6*, *PbbZIP4*, *PbERF1*, *PbMYB22*, and *PbNAC1*, showed enhanced differential expression between the two transcriptomes and were chosen for further analysis. The expression levels of these TF genes in both transcriptomes are available at Dryad (Dataset 1, ‘TF’ spreadsheet, https://doi.org/10.5061/dryad.kt056q7).

### Confirmation of the transcript levels of structural genes and TFs by quantitative real-time PCR

**Fig. 5.**
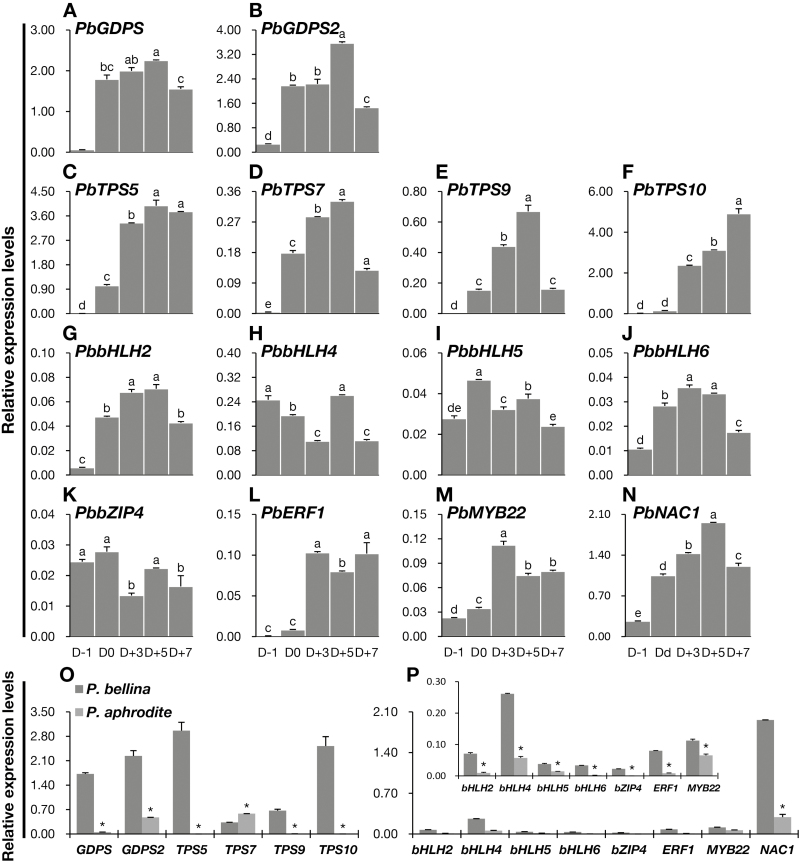
Transcript levels of structural genes and transcription factors (TFs) in *Phalaenopsis* orchids. (A–N) Expression patterns during floral developmental stages in *P. bellina* as determined by quantitative real-time PCR for six structural genes for monoterpene biosynthesis, including *PbGDPS* (A), *PbGDPS2* (B), *PbTPS5* (C), *PbTPS7* (D), *PbTPS9* (E), *PbTPS10* (F), and eight candidate TFs genes isolated by bioinformatics analysis, including *PbbHLH2* (G), *PbbHLH4* (H), *PbbHLH5* (I), *PbbHLH6* (J), *PbbZIP4* (K), *PbERF1* (L), *PbMYB22* (M), and *PbNAC1* (N). Expression was determined from the day before anthesis (D–1) to day 7 after anthesis (D+7). Pairwise comparisons between groups were performed by using Tukey’s honestly significant difference test, and different letters indicate significant differences at α=0.05. (O, P) Expression levels of six structural genes (O) and the eight candidate TFs (P) in the flowers of scentless *P. aphrodite* (at the D+5 stage) as determined by quantitative real-time PCR. Their expression levels in the scented *P. bellina* are also included for comparison. The insert in (P) shows the relatively lower expression levels of *PbbHLH2*, *PbbHLH4*, *PbbHLH5*, *PbbHLH6*, *PbbZIP4*, *PbERF1*, and *PbMYB22* in *P. aphrodite*. Statistical analysis of the expression levels of *Pa* genes and *Pb* genes was performed by using Student’s *t*-test at α=0.05. Expression was normalized to that of *Actin1*. Data are means (±SE) from three replicates.

The transcript levels of *GDPS*, *MTPS*s, and the eight candidate TFs at the various floral developmental stages in the scented *P. bellina* were confirmed by quantitative real-time PCR (Fig. 5A–N). The expression of the structural genes involved in monoterpene biosynthesis sharply increased either upon anthesis (*PbGDPS* and *PbGDPS2*) (Fig. 5A, B) or at D+3 (*PbTPS5*, *PbTPS7*, *PbTPS9*, and *PbTPS10*) (Fig. 5C–F), concomitant with the emission pattern of monoterpenes (Fig. 1C). Among the four *MTPS*s, both *PbTPS5* and *PbTPS10* were highly expressed (Fig. 5C, F) and were responsible for the production of geraniol and linalool, respectively (Y-C Chuang, unpublished results). The expression of the eight TFs either peaked on D+5 (*PbbHLH2*, *PbbHLH4*, and *PbNAC1*) or peaked on D0 or D+3 prior to the expression of the structural genes (*PbbHLH5*, *PbbHLH6*, *PbbZIP4*, *PbERF1*, and *PbMYB22*).

The expression levels of these TF genes in the scentless *P. aphrodite* were also verified and showed results that were consistent with the comparative transcriptomic analysis. *GDPS*, *TPS5*, *TPS9*, and *TPS10* were highly expressed in scented *P. bellina* but were barely detectable in scentless *P. aphrodite* (Fig. 5O). The differential expression of *GDPS2* was to slightly lesser extent than for *GDPS*, while *TPS7* showed slightly higher expression in scentless *P. aphrodite* than in scented *P. bellina.* The differential expression of the eight TFs were confirmed, with all showing higher expression in *P. bellina* (Fig. 5P).

### Transactivation of TFs on the promoters of the structural genes

**Fig. 6.**
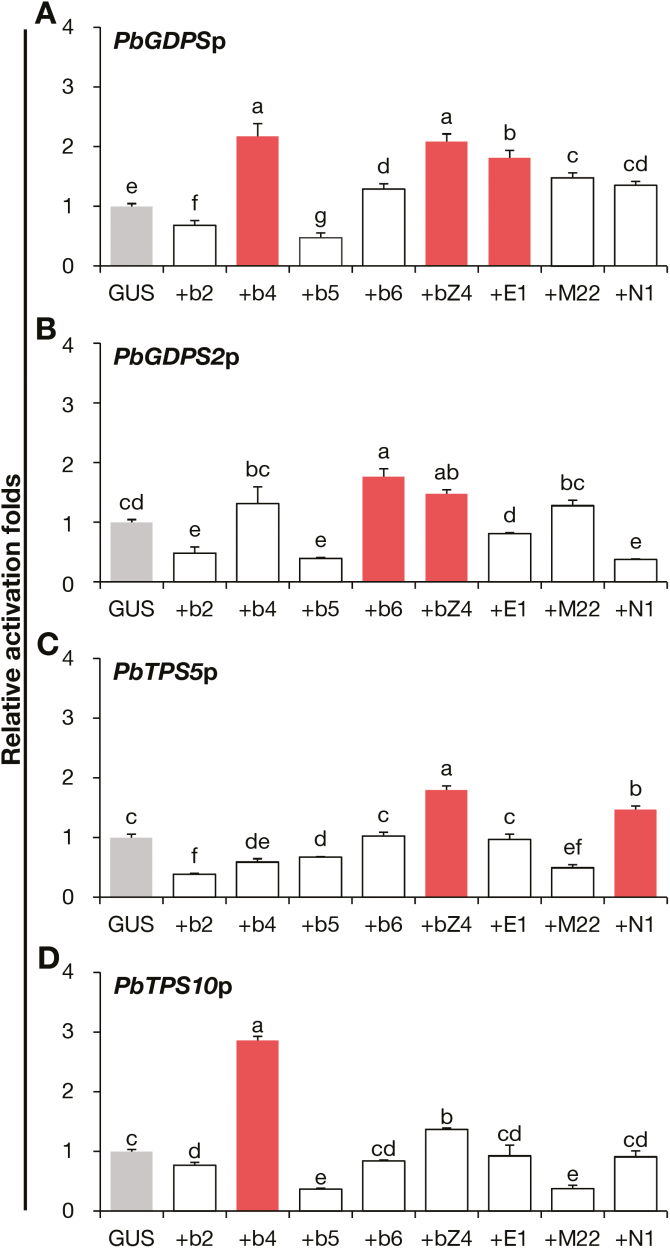
Transactivation of the eight transcription factors (TFs) on promoter fragments of four structural genes. (A–D) Transactivation activity of the eight TFs on promoter fragments of *PbGDPS* (A), *PbGDPS2* (B), *PbTPS5* (C), and *PbTPS10* (D), in *P. aphrodite* flowers by particle bombardment. The transient assays were performed with: GUS; +b2, PbbHLH2; +b4, PbbHLH4; +b5, PbbHLH5; +b6, PbbHLH6; +bZ4, PbbbZIP4; +E1, PbERF1; +M22, PbMYB22; and +N1 PbNAC1. The transactivation activity was evaluated according to relative fold-activity compared to the GUS control (grey). Activity with >1.5-fold change is highlighted in red. Data are means (±SE) of three biological replicates. Pairwise comparisons between groups were performed using Tukey’s honestly significant difference test, and different letters indicate significant differences at α=0.05.

To evaluate whether *PbGDPS* could be transactivated by the eight TFs, we used dual luciferase assays with the coding sequences of the TFs under the control of the CaMV 35S promoter. The promoter fragments of *PbGDPS2*, *PbTPS5*, and *PbTPS10* (designated *PbGDPS2*p, *PbTPS5*p, and *PbTPS10*p) were also analysed as they showed significant differential expression between *P. bellina* and *P. aphrodite*. Transient assays were performed in the perianths of the scentless *P. aphrodite* flowers, with *GUS* as a negative control. Among the four bHLHs, PbbHLH4 and PbbHLH6 transactivated *PbGDPS*p and *PbTPS10*p, and *PbGDPS2*p (Fig. 6A, B, D), but not *PbTPS5*p. PbbZIP4 enhanced the promoter activities of *PbGDPS*p, *PbGDPS2*p, and *PbTPS5*p (Fig. 6A–C), but not *PbTPS10*p. In contrast, PbERF1 only transactivated *PbGDPS*p (Fig. 6A), and PbNAC1 only transactivated *PbTPS5*p (Fig. 6C). PbbHLH2, PbbHLH5, and PbMYB22 did not transactivate any of the promoters tested, and in some cases even resulted in weak repression. Taken together, among the eight candidate TFs isolated by the bioinformatics analysis, five showed transactivation for monoterpene biosynthesis genes and acted as positive transactivators, namely PbbHLH4, PbbHLH6, PbbZIP4, PbERF1, and PbNCA1.

### Transient ectopic expression of the five candidate TFs in the scentless orchid

**Fig. 7.**
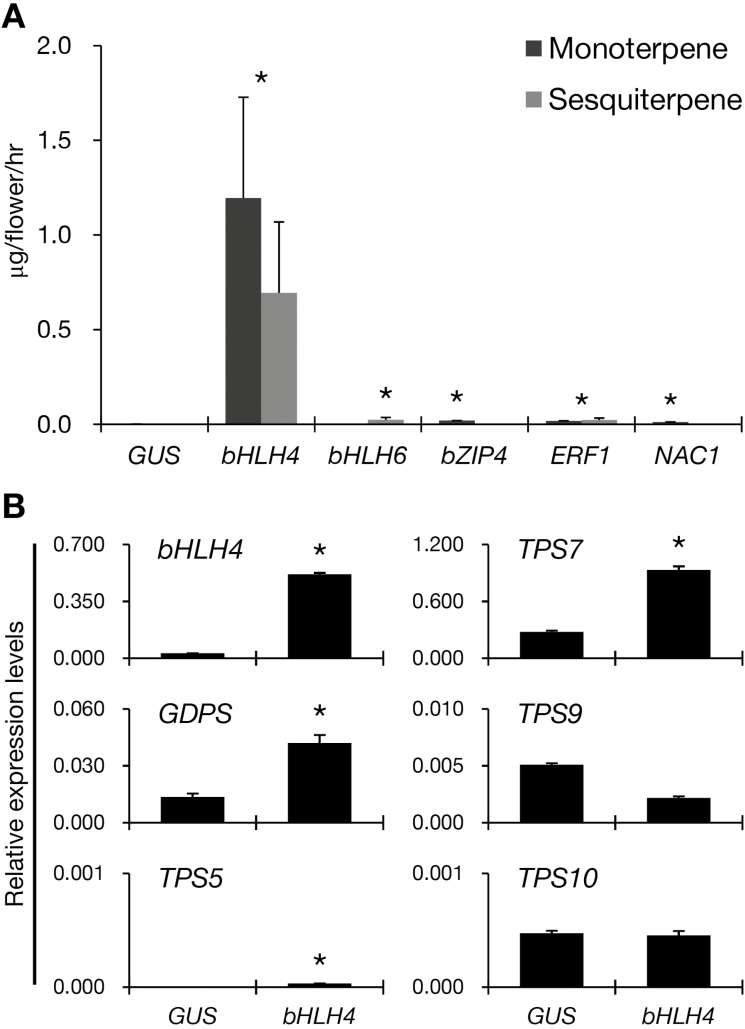
Scent compounds produced by the transient ectopic expression of transcription factors (TFs) in flowers of scentless *P. aphrodite*. (A) Content of emitted terpenes from flowers with transient ectopic expression of *GUS*, *PbbHLH4*, *PbbHLH6*, *PbbZIP4*, *PbERF1*, and *PbNAC1* was analysed at 5 d post-infiltration. *GUS* was used as a control. Data are means (±SE) from three infiltrations. (B) Expression levels of *bHLH4*, *GDPS*, and four *MTPS*s in the flowers of *PbbHLH4*-expressing *P. aphrodite* as determined by quantitative real-time PCR. Expression was normalized to that of *Actin1*. Data are means (±SE) from three replicates. Statistical analysis in comparison with the *GUS* control were performed using Student’s *t*-test at α=0.05.

The above results demonstrated that low expression of TFs in the scentless phenotype *P. aphrodite* was associated with low expression of *GDPS* and *MTPS*s. We hypothesized that ectopic expression of the five transactivators in *P. aphrodite* might induce a scented phenotype. Efficient stable transformation systems for *Phalaenopsis* orchids are lacking, with those available having low transformation efficiency as well as long regeneration times. Therefore, to test our hypothesis we used an efficient and rapid transient expression assay by utilizing *Agrobacterium* infiltration in the perianths of *P. aphrodite* (Hsu *et al.*, 2015). The coding sequences of *PbbHLH4*, *PbbHLH6*, *PbbZIP4*, *PbERF1*, and *PbNAC1* were constructed under the control of double CaMV 35S promoters and introduced into *P. aphrodite* flowers on the day of anthesis (D0). The floral scents emitted from these flowers were measured and identified using GC-MS analysis at 4 d post-infiltration. We detected a profound enhancement (~950-fold) in a group of monoterpenoid and sesquiterpenoid emissions in *PbbHLH4*-expressing *P. aphrodite* flowers as compared with the *GUS* controls (1.89 μg versus 0.002 μg h^–1^ per flower) (Fig. 7A). The monoterpenoids were dominated by terpineol derivatives, such as α-terpineol (the major component), β-terpineol, γ-terpineol, 1-terpineol, and terpinolene, together with a trace amounts of 1,4-cineole, limonene, fenchone, and camphor. Two sesquiterpenoids, α-cedrene and β-cedrene, were also detected in the infiltrated flowers. Transient expression of *PbbHLH6*, *PbbZIP4*, *PbERF1*, and *PbNAC1* also induced monoterpenoid and/or sesquiterpenoid emissions, although to a much lower extent of ~10–20-fold (Fig. 7A).

To further examine this high induction of monoterpenoids in *PbbHLH4*-expressing *P. aphrodite* flowers, we examined the expression of structural genes for monoterpene biosynthesis. Both *GDPS* and *TPS7* showed 3-fold increases in their expression, in addition to the significant increase of *PbbHLH4* transcripts in the infiltrated flowers (Fig. 7B). This suggested that of the *TPS* genes it was specifically *TPS7* that may have accounted for the great enhancement of terpineol in the *PbbHLH4*-expressing *P. aphrodite* flowers.

### Expression of *bHLH4* is concomitant with monoterpene biosynthesis in *Phalaenopsis* orchids

**Fig. 8.**
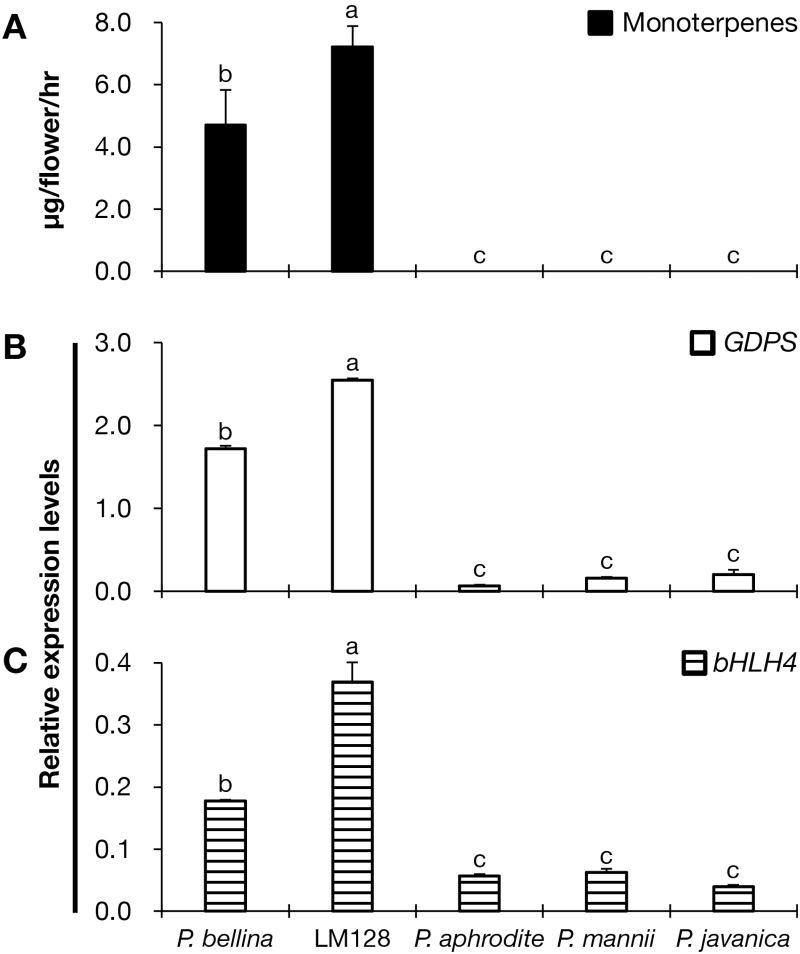
Expression patterns of *GDPS* and *bHLH4* in orchid flowers. (A) Levels of emitted monoterpenes in scented *P. bellina* (from Fig. 1B) and *P.* Meidarland Bellina Age ‘LM128’ (labeled as LM128), and scentless *P. aphrodite* (from Fig. 1B), *P. mannii* and *P. javanica* at the D+5 floral stage (5 d after anthesis). (B, C) Expression of *GDPS* (B) and *bHLH4* (C) in D+5 flowers. Expression was normalized to that of *Actin1*. Data are means (±SE) from three replicates. Pairwise comparisons between groups were performed using Tukey’s honestly significant difference test, and different letters indicate significant differences at α=0.05.

As transient expression of *PbbHLH4* resulted in monoterpene biosynthesis in *P. aphrodite*, we were interested to examine its potential role in producing a scented phenotype in other *Phalaenopsis* orchids. To this end, the expression levels of both *GDPS* and *bHLH4* were examined in other scented and scentless orchids (Fig. 8). Both genes were highly up-regulated in the commercial scented cultivar *P.* Meidarland Bellina Age ‘LM128’, an offspring of *P. bellina* that emitted high levels of monoterpenoids. In contrast, the expression levels of both genes were down-regulated in two native scentless orchids, *P. javanica* and *P. mannii*. The differential expression patterns of *GDPS* and *bHLH4* were concomitant with monoterpene production, which implied their strong association with the scented orchid phenotype.

## Discussion

### The multigene families of the MEP pathway in *P. bellina*

In this study, putative MEP pathway genes were isolated by analysing the annotation results of floral transcriptomic data, and this revealed the participation of multigene families, including *DXS*, *MCT*, *MDS*, and *HDS*, in several steps in the pathway. We noticed that in contrast to only one *MDS* gene in scentless *P. aphrodite*, seven *MDS* genes were identified in the scented *P. bellina* transcriptome (Fig. 2B). Analysis showed that these seven *PbMDS*s shared extremely high similarity in coding sequence regions, with differences of only two or three residues (Fig. S4A at Dryad). In addition, three *PbHDS* genes displayed high similarity in their sequences (Fig. S4B at Dryad). We used Trinity for *de novo* transcriptome assembly in the analysis of *P. bellina* floral transcriptomic data, which defines alternative isoforms and duplicated genes (Grabherr *et al.*, 2011). The seven detected *MDS* genes were deemed to be paralogous, but the three detected *HDS* genes were from alternative splicing. To confirm this finding, we searched for these gene sequences in the whole-genome sequence of *P. equestris* (Cai *et al.*, 2015). Six *MDS* genes distributed in six individual scaffolds were identified, but only one *HDS* gene was discovered in *P. equestris* (data not shown). Therefore, we considered *MDS* to be present in *Phalaenopsis* orchids at a high gene-copy number, but the three *HDS* genes in *P. bellina* were likely to have resulted from alternative splicing. Because the seven *PbMDS* genes had various expression patterns (Fig. 2B), we concluded that they may have distinct roles in scented orchids for differential terpenoid biosynthesis.

The whole-genome sequence of *P. aphrodite* has been released (Chao *et al.*, 2018), and we identified only one *MDS* gene within it, consistent with the result of the transcriptome analysis. This indicated that the copy number of *MDS* genes varies among *Phalaenopsis* orchids.

We identified another gene encoding geranyl diphosphate synthase, *PbGDPS2*. Its expression during the different floral developmental stages was similar to that of *PbGDPS*, with both being initiated on the day of anthesis (D0) and peaking on D+5 (Fig. 5A, B). Interestingly, *PbGDPS2* was also expressed in scentless *P. aphrodite* (Fig. 5O). In scented *P. bellina*, *PbGDPS* shows flower-specific expression (Hsiao *et al.*, 2008), while *PbGDPS2* is also expressed in leaves and roots (data from Orchidstra 2.0, see Table S3 at Dryad; Chao *et al.*, 2017). In addition to the biosynthesis of monoterpenoids, GDPS is required for biosynthesis of gibberellins in tomato, and might be involved in the biosynthesis of other terpenoids, including di-, tri-, tetra-, and/or polyterpenes (van Schie *et al.*, 2007). Thus, it is plausible that PbGDPS2 may play a different role from PbGDPS in *Phalaenopsis* orchids.

### Conserved bHLH factors for terpene regulation

The subgroup IVa of the bHLH protein family has previously been shown to be a conserved module for plant terpenoid biosynthesis regulated by jasmonate (Mertens *et al.*, 2016). Phylogenetic analysis showed that PbbHLH4 falls into the subgroup IIIb, close to the subgroup IIIe in which AtMYC2 resides, the only regulator controlling floral sesquiterpenes in the Arabidopsis inflorescence (Hong *et al.*, 2012) (Fig. S5 at Dryad). Intriguingly, AtMYC2 is also grouped with other terpene-regulation bHLHs, including AabHLH1 (Ji *et al.*, 2014), CrMYC2 (Zhang *et al.*, 2011), and SlMYC1 (Spyropoulou *et al.*, 2014*b*), indicating a well-conserved regulation of terpenoids by bHLHs in the plant kingdom.

In this study, we also isolated PbbHLH9 as being classified in the subgroup IIIe (Fig. S5 at Dryad). However, we applied two criteria to characterize the upstream factor for regulating *PbGDPS*, and PbbHLH9 was excluded because its expression pattern belonged to the STEM profile ‘5’ (Fig. S3 at Dryad) and its expression showed little differences between *P. bellina* and *P. aphrodite* (Fig. 4). Nevertheless, because most genes in the MEP pathway showed similar expression between *P. bellina* and *P. aphrodite* (Fig. 2B), the possibility of the involvement of PbbHLH9 in the regulation of floral terpenes in orchids needs to be further examined. In addition, we do not preclude the possibility that additional *bHLH* genes might play similar roles to PbbHLH4 in the regulation of terpene biosynthesis in *Phalaenopsis* orchids.

### Developmental regulation for floral monoterpene biosynthesis

For the correct the timing of a plant event, a set of internal mechanisms is often co-ordinated to occur in coincidence with, or prior to, the event proceeding. The emission of monoterpenes in *P. bellina* began on D0 (the start of blossoming), while their release strikingly increased on D+1 (by ~30-fold, Fig. 1C) as the flowers became fully opened. The crucial enzyme *PbGDPS* exhibited induction on D0 (Fig. 5A) prior to emission of volatiles, and its upstream TFs showed either much earlier expression at the big-bud stage (D–1) (*PbbHLH4* and *PbbZIP4*, Fig. 5H, K), or expression coinciding with volatile emission (*PbbERF1*, Fig. 5L). On the other hand, *PbMTPS*s, the final step of the pathway, were expressed coincidentally with the formation of volatiles (Fig. 5C, D, E, F). This well-designed regulation of the metabolic pathway contributed to monoterpene emission in the early floral stages of *P. bellina*.

The emission of monoterpenes in *P. bellina* flowers gradually increased over the first 5 d after anthesis, remained relatively steady up to D+9, and decreased thereafter (Fig. 1C). We speculate that this developmental modulation of floral scents might be designed to facilitate pollination. A study of *P.* ‘Tianxiang’ showed that the receptivity of the stigma increases after anthesis, but becomes weaker around half the life span of the flower (Yu and Li, 2017). The decreasing emission of scent with ageing might also be related to energy conservation (Dudareva *et al.*, 1999).

The phylogenetic analysis showed that PbbHLH4 was grouped with AtbHLH33/ICE2/SCRM2 and AtbHLH116/ICE1/SCRM (Fig. S5 at Dryad), which play dual roles in cold acclimation and stomatal differentiation (Kanaoka *et al.*, 2008; Kim *et al.*, 2015). Jasmonate is a critical upstream signal for an ICE-mediated cold-stress response pathway (Hu *et al.*, 2013). Moreover, jasmonate, or jasmonic acid, is a general inducer of terpenoid biosynthesis for defense responses (Martin *et al.*, 2002; Fäldt *et al.*, 2003; Miller *et al.*, 2005; Erbilgin *et al.*, 2006; Lundborg *et al.*, 2016; Yoshitomi *et al.*, 2016). Several reports have described the involvement of jasmonic acid in stress responses in *Phalaenopsis* orchids, such as enhanced cold, heat, or drought tolerance in seedlings (Huageng *et al.*, 2011; Zou *et al.*, 2011) and reduced flower-bud abortion during shipping (Chen, 2005).

Overall, PbbHLH4 may regulate the biosynthesis of floral monoterpenes in *P. bellina* in order to attract pollinators and also for stress responses, but additional studies are needed.

### Possible negative regulators for the biosynthesis of floral monoterpenes

Using transactivation assays, we found that the three TFs, namely PbbHLH2, PbbbHLH5, and PbMYB22, showed negative effects on the promoter activities of structural genes for monoterpene biosynthesis (Fig. 6), suggesting that they might repress the expression of these structural genes to some extent. Two studies in spearmint have reported repressors for monoterpene biosynthesis, MsYABBY5 and MsMYB, which were found to be highly expressed in peltate glandular trichomes where monoterpenes are accumulated (Wang *et al.*, 2016; Reddy *et al.*, 2017). It would be worthwhile studying the roles of these factors and how they interact with other activators in regulating the expression of the structural genes involved in monoterpenes biosynthesis.

### Possible reasons for low expression of *bHLH4* in *P. aphrodite*

We conclude that the lack of activators, especially bHLH4, led to the scentless phenotype in *P. aphrodite*. To examine why *bHLH4* was expressed at a low level in *P. aphrodite*, we first compared the coding sequence (CDS) of *PabHLH4* with *PbbHLH4*. Interestingly, two single-nucleotide insertions were found in the CDS of *PabHLH4*, leading to a frame-shift and a premature stop codon in the coding region (Fig. S6A at Dryad). This could be the main reason for the low expression of *PabHLH4* in *P. aphrodite*. In addition, a large insertion and numerous nucleotide substitutions were also found in the promoter fragment of *PaHLH4* (*PaHLH4*p) as compared to that of *PbHLH4*p (Fig. S6B at Dryad).

### Potential interactions of bHLH4 with other activators

We examined the expression profiles of the TFs and their transactivation activity on the various structural genes in order to determine whether there were any interactions or additive activation effects between the TFs. Previous studies have shown interactions between different classes of TFs in regulating terpene biosynthesis. For instance, CrWRKY1 is an upstream regulator for CrORCA3, CrMYC2, and CrZCTs in *C. roseus* (Pan *et al.*, 2016), and SlMYC1 together with SlEOT1 have synergistic effects that induce *SlTPS5* promoters in tomato (Spyropoulou *et al.*, 2014*b*).

To examine this in *Phalaenopsis* orchids, we performed co-infiltration experiments in *P. aphrodite* flowers by equimolar mixing with *Agobacterium* cells harboring a single TF. However, these infiltrated flowers wilted at 3–4 d post-infiltration (DPI), indicating that the floral tissue could not stand the stress of co-infiltrated. We tried diluting the bacterial solution, but there were no effects in the infiltrated flowers.

Instead, we used a *Phalaenopsis* cultivar, *P.* I-Hsin Venus, which emits a small amount of linalool (Chuang *et al.*, 2017). Although many of the infiltrated flowers also wilted at 3–4 DPI, a 2-fold induction of monoterpenes was detected in *PbbHLH4*-expressing flowers as compared to the *GUS* control (Fig. S7A at Dryad). The increased monoterpenoids included a large proportion of linalool and trace amounts of ocimene and eucalyptol. With a combination of *PbbHLH4* and *PbbZIP4*, the monoterpene level showed a 3-fold increase as compared to the *GUS* control. Gene expression analysis showed that this combination induced increases in the transcript levels of *GDPS*, *TPS5*, *TPS7*, and *TPS9* (Fig. S7B at Dryad). *PbZIP4* alone did not have any activation effects (data not shown), suggesting that there was a synergistic effect between *PbbHLH4* and *PbbZIP4*. In contrast, the combination of *PbbHLH4* and *PbNAC1* did not further enhance the levels of monoterpenes as compared to *PbbHLH4* alone (Fig. S7A at Dryad). This suggested that the interactive effects between these two TFs were not as high as *PbbHLH4* and *PbbZIP4*. Furthermore, the combination of all three TFs decreased the emission of monoterpenes as well as the expression levels of individual TFs, as compared to plants infiltrated with one or two TFs. This could have been due to dilution effects or competition between the 35S promoters for all three TFs.

Overall, the results indicate that PbbHLH4 may interact with PbbZIP4 and/or PbNAC1 in regulating monoterpene biosynthesis in *Phalaenopsis* orchids to varying degrees.

### Manipulation of floral volatile terpenoids by TFs in orchids

PbbHLH4 was able to transactivate the upstream promoters of *PbGDPS* and *PbTPS10* in transient assays (Fig. 6), which indicated that they are downstream target genes of PbbHLH4. PbGDPS is a dual-function prenyltransferase as recombinant PbGDPS yields GDP and FDP with IDP/DMADP and IDP/GDP as substrates for monoterpene and sesquiterpene production, respectively (Hsiao *et al.*, 2008). The transactivating ability of PbbHLH4 on *PbGDPS* might account for it facilitating both monoterpene and sesquiterpene biosynthesis in the transient-expression flowers. Ectopic expression of a spearmint R2R3MYB, MsMYB, whose target gene is *GDPS* affects both monoterpenes and sesquiterpenes in sweet basil (Reddy *et al.*, 2017). Moreover, ectopic transient expression of *PbbHLH4* in the scentless commercial cultivar *P.* Sogo Yukidian ‘V3’ increased the internal pools of sesquiterpenes (Fig. S8 at Dryad), so PbbHLH4 might control the metabolic flux in different branches of terpene biosynthesis.

It was interesting that the transient expression of *PbbHLH4* in the perianths of scentless *P. aphrodite* induced the production of terpineol (Fig. 7A), which has an odor similar to lilac and a sweet smell (Dionísio *et al.*, 2012). Terpineol derivatives were found at very low levels in the floral scent profile of *P. bellina* (data not shown). We speculate that the terpineol derivatives might be generated from the downstream MTPS in the scentless orchid. By analysing the expression of structural genes for monoterpene biosynthesis in the *PbbHLH4*-expressing *P. aphrodite* flowers, we found that expression of both *GDPS* and *TPS7* showed a 3-fold increase (Fig. 7B), indicating that PbbHLH4 was able to regulate *GDPS* and terpene synthase. In contrast, the expression of *PaTPS10* was not activated by PbbHLH4, which might be a result of differences in their promoter sequences (Fig. S9B at Dryad). We do not exclude the possibility that TFs other than PbbHLH4 may have played more important roles in regulating *PbTPS5* and *PbTPS10*; however, given the lack of higher *GDPS* induction, the production of linalool and geraniol was naturally at a lower level in the infiltrated flowers.

In this study, we selected *P. aphrodite* as the scentless orchid with which to make comparisons because it has overtaken many other varieties in terms of its commercialization. However, *P. aphrodite* has a small genome size (2.80 pg/2C) as compared with *P. bellina* (15.03 pg/2C) (Lin *et al.*, 2001) and there is usually cross-incompatibility between *P. bellina* and *P. aphrodite*, or its offspring. In some cases, despite successful crossing, the offspring are infertile. This is one of the obstacles faced by orchid breeders in trying to induce the scented trait into commercial cultivars with a *P. aphrodite* background. Therefore, because transient ectopic expression of *PbbHLH4* strongly induced the scent volatiles in *P. aphrodite*, it has great potential for molecular breeding of scented cultivars.

## Data Deposition

The following figures, tables and datasets are available at the Dryad Data Repository: https://doi.org/10.5061/dryad.kt056q7.

Fig. S1. The putative protein sequences of GDPS and four TPSs from both *P. bellina* and *P. aphrodite*.

Fig. S2. Phylogenetic tree of the putative PbMTPS.

Fig. S3. Gene cluster analysis of transcription factors as determined using the STEM software.

Fig. S4. Multiple alignment of the MEP pathway gene families in the *P. bellina*

transcriptome.

Fig. S5. Phylogenetic tree inferred from the amino sequences of PbbHLH4 with terpene-related bHLHs and the IIIb and IIIe subgroups of Arabidopsis bHLHs.

Fig. S6. Multiple alignment of the coding region and promoter fragments of *PabHLH4* and *PbbHLH4*.

Fig. S7. Scent compounds produced by the transient ectopic expression of *PbbHLH4* with *PbbZIP4* and/or *PbNAC1* in flowers of *P.* I-Hsin Venus.

Fig. S8. Scent compounds produced by the transient ectopic expression of *PbbHLH4* in flowers of *P.* Sogo Yukidian ‘V3’.

Fig. S9. Multiple alignment of the promoter fragments of *TPS5* and *TPS10* isolated from *P. aphrodite* and *P. bellina*.

Table S1. TFs involved in the regulation of terpene biosynthesis.

Table S2. Sequences of all primers used in this study.

Table S3. Expression levels of *GDPS* and *GDPS2* in vegetative organs in *P. bellina*.

Dataset 1. Expression levels of reference, structural, and TF genes in the two transcriptomes examined.

Dataset 2. STEM clustering results for associating expression patterns of the transcription factors examined.
